# Reliable Atom Probe Quantification of Carbon in Non-Stoichiometric TiC_x_: Effects of Laser Energy and Correction of Carbon Loss

**DOI:** 10.3390/ma19194055

**Published:** 2026-09-23

**Authors:** Yibing Xie, Haokai Su, Bing Hu, Shenbao Jin

**Affiliations:** 1 School of Materials Science and Engineering, Hebei University of Technology, Tianjin 300401, China; 2 State Key Laboratory of High Performance Roll Materials and Composite Forming, Hebei University of Technology, Tianjin 300401, China; 3 School of Materials Science and Engineering, Nanjing University of Science and Technology, Nanjing 210094, China; 4 Sinosteel Xingtai Machinery & Mill Roll Co., Ltd., Xingtai 054099, China

**Keywords:** TiC_x_, atom probe tomography, carbon quantification, laser energy

## Abstract

Accurate carbon quantification in TiC_x_ by atom probe tomography (APT) is strongly affected by the evaporation of carbon-containing molecular ions, detector dead time and peak overlap. Here, APT mass spectra, hit multiplicity and correlation histograms acquired at 40–400 pJ were analyzed to characterize laser-energy-dependent evaporation. With increasing laser energy, carbon shifted toward C_2_^+^–C_5_^+^ molecular ions and Ti-C complexes, including TiC^+^, TiC_2_^+^–TiC_5_^+^ and Ti_2_C_2_^2+^. The fraction of detected carbon carried by molecular ions increased from 47% to 98%, while the uncorrected C/Ti ratio decreased from 0.52 to 0.26. Multiplicity and correlation analyses indicated that carbon-rich molecular ions were susceptible to incomplete registration within the detector dead time. After correction for dead-time-related losses of C^2+^ and C^+^ at 6 and 12 Da as well as for the 24 Da overlap between ^48^Ti^2+^ and C_2_^+^, the C/Ti ratio increased to 0.92 at 40 pJ and 0.83 at 60 pJ, approaching the XRD-derived range of 0.85–0.95. At higher energies, the correction remained insufficient because the method does not explicitly account for C_3_^+^–C_5_^+^ molecular-ion losses, whose broad peaks and unresolved minor-isotope peaks prevent reliable isotope-based correction. Therefore, among the investigated conditions, 40 pJ is preferable for reliable APT carbon quantification in TiC_x_.

## 1. Introduction

Titanium carbide (TiC) has high hardness, high elastic modulus, high flexural strength, good corrosion resistance and low density, making it widely used as a surface coating material and as a reinforcing phase in metal–matrix composites [1,2,3,4,5]. Stoichiometric TiC has a face-centered cubic NaCl-type structure with equal numbers of Ti and C atoms. However, TiC usually exists as non-stoichiometric TiC_x_ owing to the presence of carbon vacancies, with x varying from 0.47 to 0.98 while maintaining the NaCl-type structure [6]. The C/Ti ratio strongly affects the bonding state and mechanical properties of TiC_x_, such as hardness and elastic modulus [7,8,9,10]. It can also influence the growth morphology of TiC_x_ particles [11,12,13,14] and the interfacial bonding [15,16,17] between TiC_x_ and the matrix in metal-based composites. Therefore, accurate determination of the C/Ti ratio is essential for understanding the structure–property relationship of non-stoichiometric TiC_x_.

Accurate carbon quantification of TiC_x_ is difficult using conventional characterization methods. Energy-dispersive X-ray spectroscopy is not sufficiently reliable for light elements, while combustion-based carbon analysis may suffer from sampling and scaling errors for materials with high carbon contents. X-ray diffraction (XRD) provides an indirect method for estimating TiC_x_ stoichiometry through the lattice-parameter and C/Ti ratio relationship [18,19,20]. However, the lattice parameter can also be affected by residual stress, impurity incorporation and synthesis conditions. Therefore, although XRD-derived stoichiometry can provide a useful reference, it cannot be regarded as an absolute measurement of TiC_x_ composition.

Atom probe tomography (APT), based on time-of-flight mass spectrometry, enables high-sensitivity three-dimensional chemical mapping at near-atomic resolution. It is therefore a promising technique for directly quantifying the TiC_x_ stoichiometry. Nevertheless, quantitative carbon analysis in carbides by APT remains challenging. Carbon can be detected as monatomic ions, carbon molecular ions and Ti-C complex ions, leading to peak overlap and peak-assignment uncertainty [21,22]. Moreover, carbon-containing ions are frequently involved in multiple-hit and correlated evaporation events, where detector dead time or ion-pile-up effects may cause preferential carbon loss and underestimation of the C/Ti ratio [23].

Recent studies have considerably advanced the understanding of carbon field-evaporation behavior in carbides during APT. In SiC, Ndiaye et al. found that ~84% of the detected carbon ions occurred in multiple-hit events [24]. The measured carbon content was ~46 at.% rather than the nominal 50 at.%, and carbon was affected by detector pile-up approximately twice as strongly as silicon. Using reflectron-equipped and straight-flight-path instruments, Schiester et al. measured only 39.0 and 33.8 at.% C in TiC, respectively, compared with the independently determined value of ~50.7 at.% [25]. Their out-of-sync correlation analysis further showed that carbon was involved in almost all identified delayed TiC ion pairs, revealing an additional source of carbon undercounting. Schreber et al. subsequently discussed the use of correlation-histogram and background-recovery analyses for identifying delayed evaporation events in oxides, nitrides and borides [26]. In a systematic study of a TiN-Ti(C,N)-TiC coating series, Schiester reported a pronounced dependence of the measured TiC composition on laser pulse energy [27]. The study primarily addressed acquisition-induced compositional deviations and identified ion pile-up and molecular dissociation. However, quantitative evaluations of the effectiveness and limitations of carbon-loss corrections over a broad laser-energy range, particularly for non-stoichiometric TiC_x_, remain limited. Since laser pulse energy strongly affects molecular-ion formation and correlated evaporation behavior, its influence on carbon quantification in TiC_x_ during APT needs to be clarified.

Specifically, it remains insufficiently established how laser pulse energy changes the distribution of detected carbon among monatomic ions, carbon molecular ions and Ti-C complex ions; how this redistribution influences carbon undercounting; and over which laser-energy range isotope-abundance and 24 Da peak-overlap corrections [28] remain effective for non-stoichiometric TiC_x_. Accordingly, in this work, XRD was used to derive a reference range for TiC_x_ stoichiometry, while APT datasets were acquired at 40–400 pJ. The objectives were as follows: (1) to characterize the effects of laser pulse energy on carbon-ion speciation and correlated evaporation; (2) to determine how these changes influence the measured C/Ti ratio; and (3) to evaluate the effectiveness and limitations of isotope-abundance and 24 Da peak-overlap corrections.

## 2. Experimental Procedures

The TiC_x_ target specimen was first characterized by XRD using Cu Kα radiation with a wavelength of 0.15406 nm. Phase identification was performed in continuous scanning mode over a diffraction angle range of 20–80° at a scanning rate of 2° min^–1^. Subsequently, fine XRD scans were conducted around the TiC_x_ diffraction peaks under the step scan mode with a step size of 0.02° in 2θ and a counting time of 1 s per step, to accurately determine the peak positions. Each diffraction peak was fitted independently using both Gaussian and pseudo-Voigt line-shape functions with a linear background. The fitting was repeated over slightly varied fitting windows. The peak-position uncertainty was calculated as the sample standard deviation of the fitted peak centers obtained from all combinations of the two line-shape functions and the different fitting windows. The lattice parameter was calculated from the fitted peak positions using Bragg’s law and the cubic lattice relationship, followed by Nelson–Riley extrapolation. The stoichiometry of the present TiC_x_ target was estimated based on the fitted peak positions and the reported relationship between the lattice parameter and stoichiometry of TiC_x_ [29].

APT tip specimens were prepared using the focused ion beam (FIB) lift-out method on an FEI Helios 5 UX system (Thermo Fisher Scientific, Waltham, MA, USA). APT experiments were conducted using a LEAP 4000 Si instrument (CAMECA, Madison, WI, USA) at a base temperature of 40 K in laser-pulsing mode, with a pulse repetition rate of 200 kHz. To evaluate the effect of laser energy on carbon quantification, datasets were collected at laser pulse energies of 40, 60, 80, 100, 120, 140, 200 and 400 pJ. Here, the laser pulse energy of 40 pJ was chosen as the lower bound to reduce the risk of specimen fracture while ensuring stable data acquisition. The upper bound of 400 pJ was deliberately included to extend the investigated range and more clearly reveal the laser-energy-dependent evolution of molecular-ion formation and carbon loss. One independently prepared APT tip was analyzed at each laser pulse energy. Each dataset contained 5–6 million detected ions, providing sufficient counting statistics for evaluating the C/Ti ratio and the relative abundances of the detected ion species. Data reconstruction and analysis were performed using CAMECA IVAS 6.3. The same peak-ranging strategy was applied to all datasets to minimize artificial differences caused by inconsistent mass-spectrum interpretation. More detailed information about APT data can be found in Table S1, Supplementary Materials.

## 3. Results and Discussion

### 3.1. XRD Characterization and Reference Composition

Figure 1 shows the XRD pattern and lattice-parameter extrapolation of the TiC_x_ specimen. The diffraction pattern agrees well with the face-centered cubic TiC phase (PDF #32-1383), and no obvious impurity phases were detected. Five diffraction peaks corresponding to the (111), (200), (220), (311) and (222) planes were identified. Based on the fine-scan data and peak-profile fitting, the peak positions were determined to be 2θ = 36.080°, 41.869°, 60.611°, 72.482° and 76.250°, respectively. The average uncertainty of the fitted peak positions was ±0.002° in 2θ.

The extrapolated lattice parameter was ~0.4329 nm (Figure 1b). According to the relationship between the TiC_x_ lattice parameter and stoichiometry in [29], the estimated C/Ti ratio of the specimen was ~0.85–0.95. Because the lattice parameter can also be affected by residual stress, impurity incorporation and instrumental errors, this XRD-derived value was used as a reference for evaluating the reliability of APT-based carbon quantification rather than as an absolute composition standard.

### 3.2. APT Analysis

#### 3.2.1. Mass Spectra and Carbon-Containing Ion Species

Figure 2 shows typical APT mass spectra of the TiC_x_ specimen obtained at different laser pulse energies. As shown in Figure 2a, at 40 pJ, carbon-related peaks, including C^+^, C^2+^, C_3_^+^, C_4_^+^, C_5_^+^ and TiC^+^, can be clearly identified, together with Ti-related peaks. Minor O-, Al- and N-containing peaks were also detected, but their intensities were very low and did not affect the subsequent carbon quantification. Considering the presence of higher-order carbon molecular ions (C_3_^+^, C_4_^+^, C_5_^+^), C_2_^+^ species are also expected; however, C_2_^+^ cannot be directly ranged because it completely overlaps with Ti^2+^ at 24 Da. When the laser pulse energy was increased from 40 to 200 pJ, the main ion species observed in the mass spectra remained similar, as shown in Figure 2b and Figure S1. In contrast, when the laser energy was further increased to 400 pJ, the mass spectrum became much more complex. A large number of complex Ti-C molecular ions such as TiC_2_^+^, TiC_3_^+^, TiC_4_^+^, TiC_5_^+^ and Ti_2_C_2_^2+^ were detected, indicating that excessive laser energy strongly promotes the formation and evaporation of complex molecular ions.

The formation of complex carbon-containing molecular ions increases the possibility of peak overlap in the mass spectrum. For example, C_2_^+^ overlaps with Ti^2+^ at 24 Da, C_4_^+^ overlaps with Ti^+^ at 48 Da, and C_5_^+^ can overlap with Ti_2_C_2_^2+^ at 60 Da. If these overlapped peaks are simply assigned to Ti-containing ions, part of the carbon signal will be missed, leading to an underestimation of the carbon concentration. Second, molecular-ion evaporation is often associated with correlated evaporation and multiple-hit events. In such events, two or more ions are evaporated and detected within the same pulse. Carbon-containing ions are therefore more likely to be affected by detector dead time. When multiple ions generated by the same pulse arrive at the detector within a very short time interval or within a small detector area, their signals may not be fully resolved due to the finite detector response time and local ion pile-up [30,31]. As a result, a fraction of ions in multiple-hit events may be missed or incorrectly recorded. This can cause preferential carbon loss and thus decrease the measured C/Ti ratio.

Figure 3a shows the relative fractions of single-hit and multiple-hit events as a function of laser pulse energy. Single-hit events dominate under all testing conditions. With increasing laser energy, the fraction of single-hit events increases monotonically, whereas the detected multiple-hit fraction decreases. In general, a higher single-hit fraction is considered favorable for composition quantification because it reduces the probability of detector dead-time effect. However, carbon quantification in TiC_x_ is more complicated. Detector dead-time effects are not only related to the number of multiple-hit events, but also to the ion species involved in each event. A striking difference is observed between the C/Ti ratios in the single-hit and multiple-hit spectra, as shown in Figure 3b. The single-hit spectra are highly Ti-rich, whereas the multiple-hit spectra are highly C-rich. This indicates that carbon-containing ions preferentially participate in correlated evaporation events. Therefore, even though the detected multiple-hit fraction decreases with increasing laser energy, carbon-related evaporation may still be strongly affected by detector dead-time effects.

Figure 3c shows the contributions of monatomic carbon ions, including C^+^ and C^2+^, and carbon molecular ions, such as C_3_^+^, C_4_^+^ and C_5_^+^, to the total detected carbon atoms. At 40 pJ, monatomic carbon ions contribute slightly more carbon than molecular ions. However, when the laser energy exceeds 40 pJ, carbon molecular ions become the dominant contributor to the detected carbon signal, and their contribution increases continuously with increasing laser energy. Figure 3d further shows the relative fractions of different carbon evaporation forms as a function of laser energy. The fraction of carbon detected as molecular ions, mainly C_3_^+^, C_4_^+^ and C_5_^+^, increases with increasing laser energy, whereas the fractions of monatomic carbon ions and TiC ions decrease.

These results indicate that, at higher laser energies, carbon is more likely to evaporate as carbon-rich molecular ions such as C_3_^+^, C_4_^+^ and C_5_^+^. Therefore, even if the number of detected multiple-hit events decreases, the total amount of carbon involved in molecular-ion evaporation and potentially affected by detector dead time effect may still increase. In other words, the detected multiple-hit fraction reflects the number of detected multi-ion events, whereas carbon loss depends on the number of carbon atoms contained in these events. This explains why a higher single-hit fraction at elevated laser energy does not necessarily lead to more reliable carbon quantification in TiC_x_.

#### 3.2.2. Multiple-Hit and Correlated Evaporation Behavior

Correlation histogram analysis was further performed to examine ion-pair correlations within multiple-hit events. As demonstrated by Saxey [32], correlation histograms can reveal non-random correlations between ions generated within the same pulse and provide insight into spectrum noise, molecular-ion dissociation and possible data loss caused by ion pile-up. In the present TiC_x_ specimen, the correlation histograms in Figure 4 show pronounced ion-pair correlations at carbon-related and Ti-related mass-to-charge ratios. Clear horizontal and vertical features are observed around low-mass carbon peaks, indicating that carbon-containing ions are frequently involved in correlated evaporation. The strong correlation features near 24 Da are particularly important because this region contains overlapping contributions from Ti^2+^ and carbon-containing molecular ions. Although the exact carbon fraction in this peak cannot be quantified solely from the correlation histogram, these features indicate that the 24 Da peak should not be assigned only to Ti^2+^ without correction.

With increasing laser pulse energy, the correlation features become more diffuse and complex, especially in the carbon-related mass range. This behavior indicates that higher laser energy promotes more complicated evaporation pathways involving carbon molecular ions and Ti-C complex ions. Such enhanced molecular-ion and correlated evaporation can increase peak-overlap uncertainty and aggravate carbon loss through detector dead-time or ion-pile-up effects. Therefore, correlation histogram analysis provides mechanistic support for the interpretation that high laser energy increases carbon evaporation complexity.

#### 3.2.3. C/Ti Quantification and Carbon-Loss Correction

The compositions obtained by direct APT analysis under different laser pulse energies are listed in Table 1. The C/Ti ratio measured at 40 pJ is the highest among all laser energies, approximately 0.52, but it is still much lower than the XRD-derived reference value of ~0.85–0.95. With increasing laser pulse energy, the measured C/Ti ratio decreases considerably, reaching ~0.27 at 200 pJ and 0.26 at 400 pJ. These results indicate that carbon is systematically underestimated in direct APT analysis of TiC_x_.

The decrease in the measured C/Ti ratio from 0.52 at 40 pJ to 0.26 at 400 pJ was accompanied by an increase in the fraction of detected carbon carried by molecular ions from ~47% to 98%. This trend is consistent with laser-energy-dependent carbon undercounting reported for other carbon-containing materials. Kitaguchi et al. found that the apparent carbon content of cementite decreased below its stoichiometric value at higher laser energies, while larger carbon molecular ions increasingly occurred in multiple-detection events [33]. Similarly, Vogel et al. obtained compositions closer to the reference value for Hf(C,N) at lower laser energies and showed that conventional indicators such as overall hit multiplicity and mass resolving power were insufficient for assessing compositional accuracy [34]. These observations support the present interpretation that higher laser energy promotes the formation of carbon-rich molecular ions and increases their susceptibility to incomplete registration during correlated evaporation.

Nevertheless, the relationship between laser energy, multiple-hit events and carbon-quantification accuracy is not universal. Previous studies of WC and other carbide systems have shown that the measured carbon content also depends on detector characteristics, specimen temperature and local evaporation conditions [23,35,36]. Related studies on TiN further demonstrated that laser wavelength, laser pulse energy and detector performance can strongly affect molecular-ion formation and the quantitative analysis of light elements in transition-metal compounds [37,38]. Moreover, the loss of a single carbon-rich molecular ion or correlated event may correspond to several carbon atoms, even when the overall multiple-hit fraction does not increase or even decreases. Therefore, the identities of the ions and the number of carbon atoms carried by each correlated event are more informative than the total multiple-hit fraction alone. Although peak overlap, molecular-ion dissociation and background assignment may also contribute, the simultaneous increase in carbon-rich molecular ions and decrease in the measured C/Ti ratio indicates that incomplete registration of correlated carbon-containing ions should be the major contributor to the observed carbon undercounting.

Following correction strategies proposed for Ti(C,N) analysis by Angseryd et al. [28], the carbon concentration was corrected using an in-house program (see Supplementary Note S1 for the detailed equations and parameters). First, the ion counts at 6 Da and 12 Da were corrected according to the natural abundance ratio of ^12^C to ^13^C, hereafter referred to as the ^13^C correction. This correction accounts for the preferential loss of major carbon isotope signals caused by detector dead-time effects. Second, the peak overlap at 24 Da was corrected by decomposing the contribution from Ti^2+^ and C_2_^+^ ions, hereafter referred to as the 24 Da correction. Figure 5 compares the uncorrected and corrected C/Ti ratios. The C/Ti ratio increases significantly after the ^13^C correction (as indicated by the green arrows in Figure 5), demonstrating that a considerable fraction of the carbon signal is lost from the major carbon peaks due to detector-dead-time-related effects. After further correction of the 24 Da region, the C/Ti ratio increases slightly. This indicates that the influence of C_2_^+^/Ti^2+^ overlap on carbon quantification is comparatively smaller than the dead-time-related carbon loss reflected by the ^13^C correction.

It is worth noting that, after the combined ^13^C and 24 Da corrections, the C/Ti ratios obtained at 40–60 pJ approached the XRD-derived reference value. However, when the laser energy exceeded 60 pJ, the corrected values remained markedly lower. This suggests that part of the carbon signal is lost through more complex mechanisms, such as excessive molecular-ion formation and correlated evaporation, and cannot be fully recovered by the present correction procedure. In a previous APT study of combustion-synthesized TiC_x_ powders measured at 40 K and 100 pJ, the corrected C/Ti ratios were 0.71, 0.74 and 0.58, which were consistently lower than the corresponding XRD-derived values of 0.83, 0.85 and 0.79 [29]. The present results suggest that carbon quantification in TiC_x_ is highly sensitive to laser energy and that 100 pJ may not be sufficiently low to avoid laser-energy-induced carbon loss in TiC_x_. Therefore, high laser energy tends to underestimate the carbon content of TiC_x_ even after ^13^C and 24 Da corrections.

After the combined isotope-abundance and 24 Da peak-overlap corrections, the C/Ti ratios obtained at 40 and 60 pJ increased to 0.92 and 0.83, respectively, approaching the XRD-derived range of 0.85–0.95. Among the investigated conditions, 40 pJ provided the most reliable carbon quantification because its corrected C/Ti ratio fell within the XRD-derived range, whereas the value obtained at 60 pJ remained slightly below this range. Within the investigated range, lower laser pulse energies suppressed the formation and associated loss of higher-order carbon-rich molecular ions and were therefore more favorable for accurate carbon quantification. However, excessively low laser energies require a higher standing voltage to maintain evaporation, thereby increasing the risk of premature specimen fracture. Consequently, 40 pJ is considered to provide the best practical balance between carbon-quantification accuracy and specimen stability under the present experimental conditions. It is worth mentioning that the agreement obtained at 40 pJ should be interpreted as consistency between the local APT measurement and the bulk XRD-derived reference range, rather than as independent confirmation of the absolute TiC_x_ composition. Nevertheless, this agreement indicates that low-energy APT combined with the two corrections can complement XRD when localized stoichiometric information is required. In addition, because only one APT tip was analyzed at each laser-energy condition, tip-to-tip reproducibility could not be quantified. Therefore, the recommended laser pulse energy, identified as 40 pJ under the present experimental conditions, may vary with specimen and instrument conditions.

The ^13^C correction mainly accounts for dead-time-related losses of C^2+^ and C^+^ ions at 6 and 12 Da, respectively, and it does not explicitly correct the loss of carbon-rich molecular ions. As shown in Figure 3c,d, carbon is increasingly detected as molecular ions at higher laser energies, while Figure 3b and Figure 4 show that carbon-containing ions are closely associated with multiple-hit and correlated evaporation events. Therefore, the combined effect of molecular-ion evaporation and detector dead time is likely a major reason for the severe carbon underestimation at high laser energies. In principle, dead-time-related loss of molecular ions could also be evaluated using isotope-abundance considerations. In practice, however, the C_3_^+^, C_4_^+^ and C_5_^+^ peaks are broad and their minor isotope peaks are difficult to separate, making reliable correction of molecular ion loss challenging. A reflectron-equipped APT instrument with higher mass-resolving power may help reduce this uncertainty. Nevertheless, the present results show that TiC_x_ stoichiometry can be estimated more reliably by APT when low laser energy is used to suppress higher-order molecular ion loss, combined with ^13^C and 24 Da corrections.

Here, the error bars in Figure 5 represent only the uncertainty propagated from the reported ^13^C natural abundance, rather than the total analytical uncertainty. Peak ranging involves analyst judgment, and its uncertainty was not explicitly quantified. Moreover, the 24 Da peak may contain C_4_^2+^ in addition to C_2_^+^ and ^48^Ti^2+^. Assigning the recovered carbon signal entirely to C_2_^+^ would underestimate the recovered carbon if C_4_^2+^ also contributes. These overlapping contributions cannot be reliably separated from the present data, and the associated uncertainty was therefore not included in the error bars. Improved separation and quantification of these contributions warrant further investigation.

Moreover, the measured C/Ti ratio is a relative quantity, and its decrease may result from carbon undercounting, apparent Ti overestimation or both. The increased fraction of carbon carried by molecular ions, the enrichment of carbon in multiple-hit events, the pronounced carbon-related correlations and the recovery of carbon by the ^13^C correction collectively indicate that preferential carbon undercounting is the major contributor. Nevertheless, Ti-related effects cannot be completely excluded because changes in the Ti charge-state distribution and the assignment of overlapped Ti-C peaks may affect the denominator. The ^13^C correction mainly estimates losses of C^2+^ and C^+^ at 6 and 12 Da, while the 24 Da correction partially compensates for missing carbon under the assumption that the recovered signal corresponds to C_2_^+^. Neither correction explicitly accounts for losses of C_3_^+^–C_5_^+^ molecular ions, whose broad peaks and poorly resolved minor-isotope peaks hinder reliable isotope-based correction. Consequently, the corrected compositions at higher laser energies represent partial compensation for carbon loss and may still underestimate the carbon content. The individual contributions of carbon loss and Ti-related peak-assignment bias cannot be fully separated from the present data.

The present results indicate that lower laser pulse energies can suppress the formation of higher-order carbon-rich molecular ions and thereby reduce their susceptibility to incomplete detector registration. Correlation-histogram and background-recovery analyses can help identify carbon-containing ions involved in correlated or delayed evaporation events [26], while isotope-abundance and peak-overlap corrections can partially compensate for the resulting compositional bias. However, ions that are not registered cannot be fully recovered by post-processing. A recent perspective on APT measurement science similarly emphasized that molecular-ion formation, peak overlap, detector dead time, multiple-hit detection and instrument-dependent acquisition conditions remain important sources of uncertainty in quantitative compositional analysis [39]. Hardware-based approaches have also been explored. In a recent study, a grid was placed behind the local electrode, reducing the effective detection efficiency from 52% to 7% and the multiple-hit fraction in the carbide region from 15.7% to 5.0% [36]. Although this approach reduces ion-collection efficiency and increases acquisition time, it demonstrates that improving detector multi-hit capability and ion-arrival discrimination provides a promising route toward more accurate carbon quantification.

## 4. Conclusions

In this work, the effect of laser pulse energy on carbon evaporation behavior and APT quantification in non-stoichiometric TiC_x_ was systematically investigated. The main conclusions are as follows:

(1) Increasing the laser pulse energy from 40 to 400 pJ promoted the formation of C_2_^+^–C_5_^+^ molecular ions and Ti–C complex ions. Consequently, the fraction of detected carbon carried by molecular ions increased from ~47% to 98%, while the uncorrected C/Ti ratio decreased from 0.52 to 0.26.
(2) Multiplicity and correlation analyses showed that carbon-containing ions were closely associated with correlated evaporation events. The increasing number of carbon atoms carried by higher-order molecular ions makes their incomplete registration a major contributor to carbon undercounting.
(3) After isotope-abundance and 24 Da peak-overlap corrections, the C/Ti ratios increased to 0.92 at 40 pJ and 0.83 at 60 pJ, compared with the XRD-derived range of 0.85–0.95. Among the investigated conditions, 40 pJ provided the most reliable balance between carbon-quantification accuracy and specimen stability.

The present correction method remains limited at higher laser energies because it does not explicitly recover losses of molecular ions, whose broad peaks and unresolved minor-isotope peaks prevent reliable isotope-based correction. Future improvements in mass resolving power and detector multi-hit capability, together with a systematic evaluation of laser wavelength, specimen temperature and lower-energy operating conditions, should further improve the reliability of carbon quantification in carbides.

## Figures and Tables

**Figure 1:**
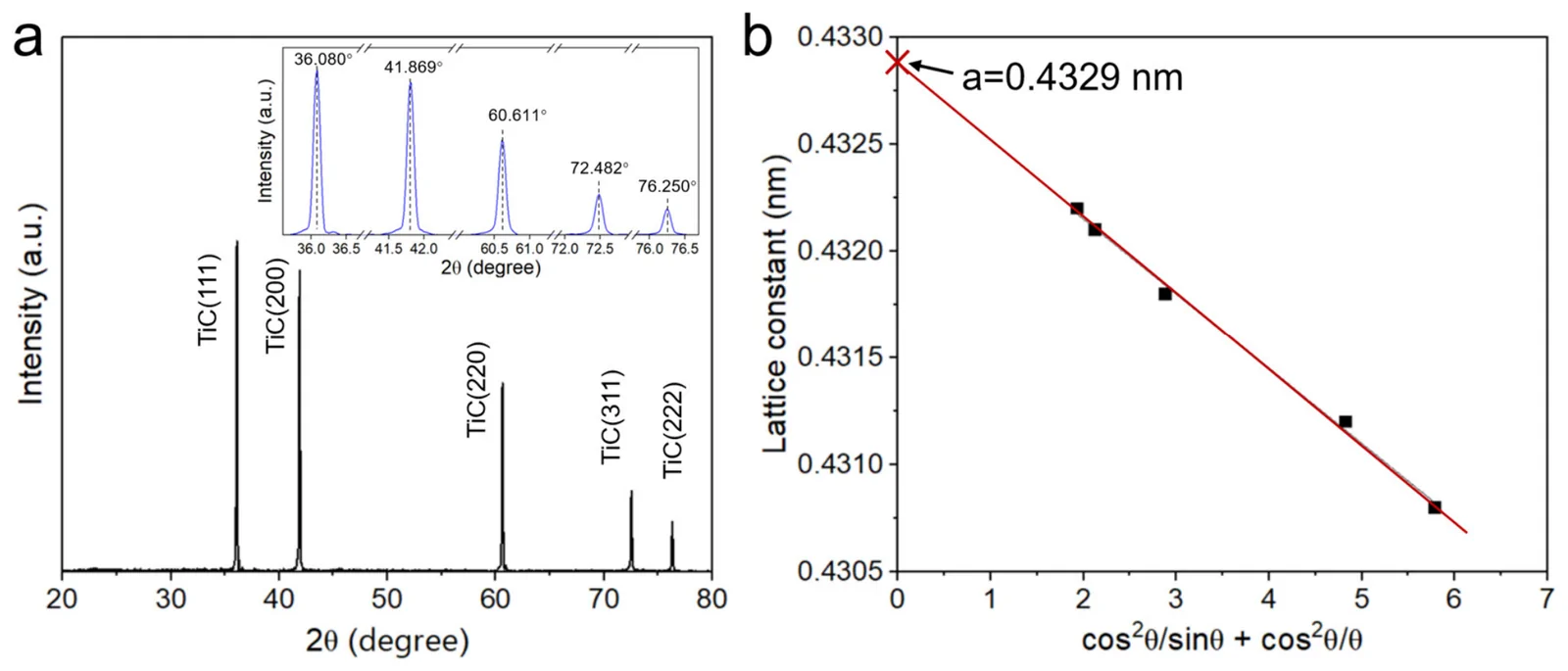
(**a**) XRD pattern of the TiC_x_ specimen with the enlarged inset showing the fitted TiC_x_ diffraction peaks. (**b**) Lattice-parameter extrapolation used to estimate the stoichiometry of TiC_x_.

**Figure 2:**
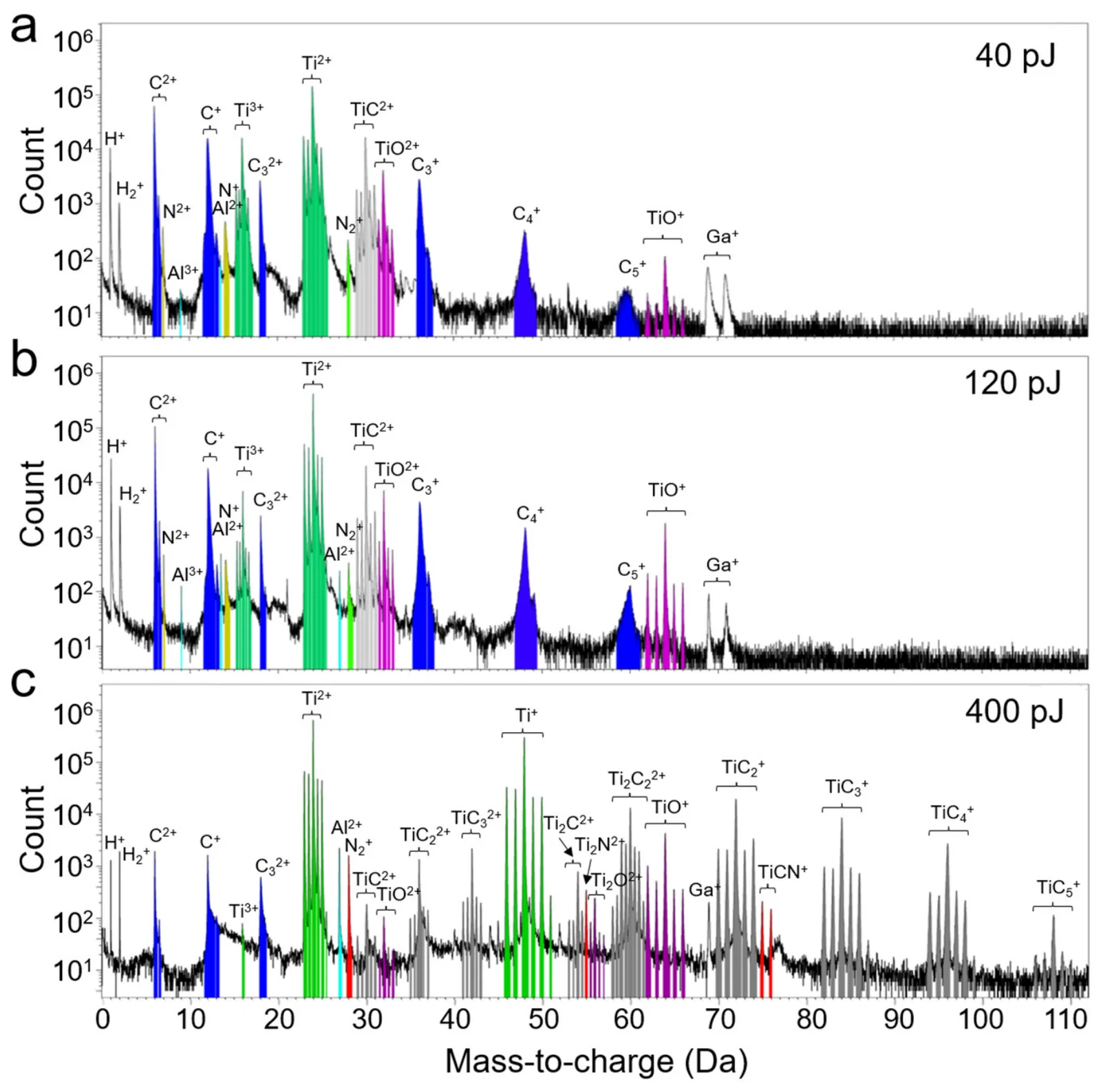
Typical APT mass spectra of TiC_x_ obtained at different laser pulse energies: (**a**) 40 pJ, (**b**) 120 pJ and (**c**) 400 pJ.

**Figure 3:**
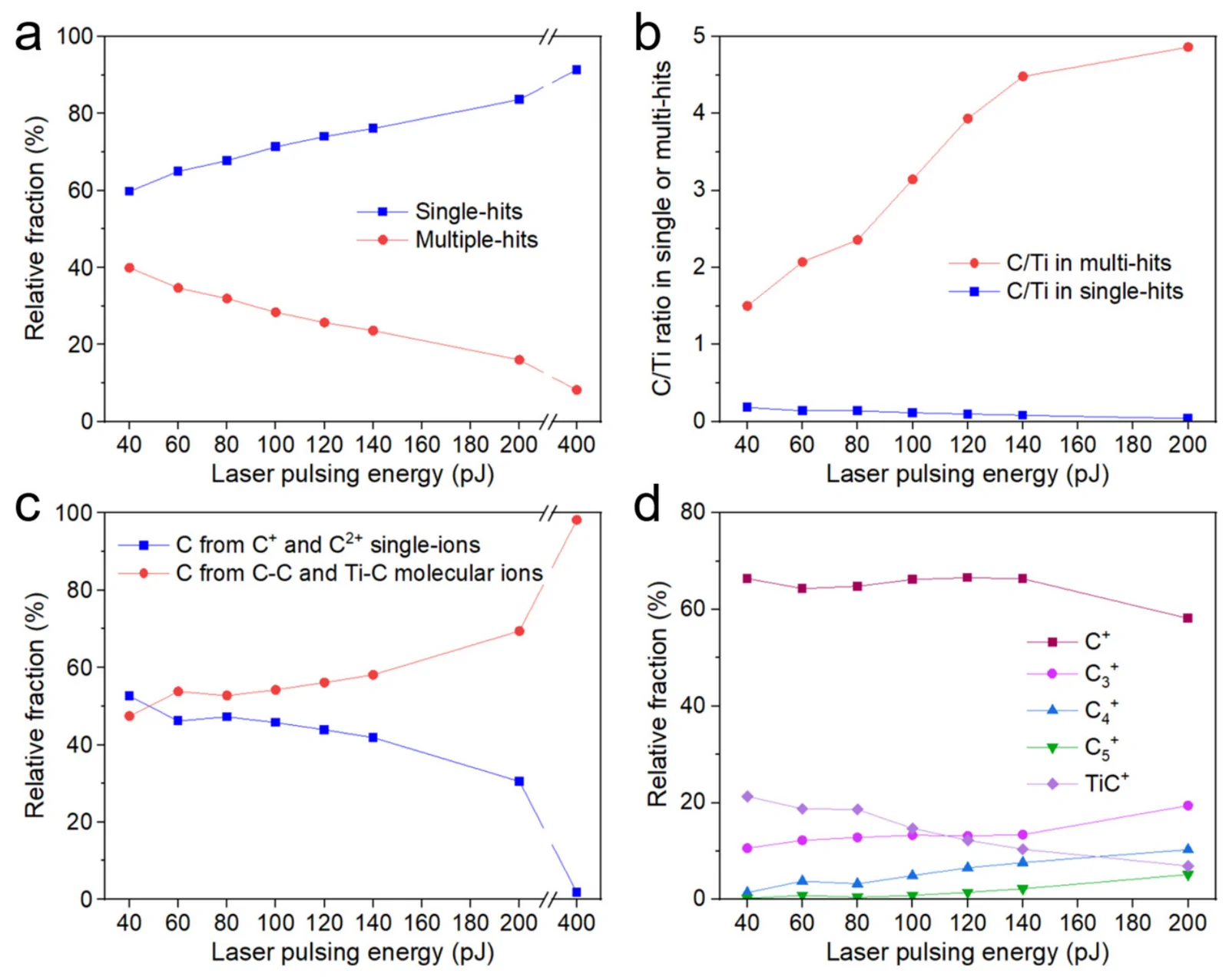
(**a**) Relative fractions of single-hit and multiple-hit events as a function of laser pulse energy. (**b**) C/Ti ratios calculated from the single-hit and multiple-hit spectra. (**c**) Contributions of monatomic carbon ions and molecular ions to the total detected carbon atoms. (**d**) Relative fractions of different carbon evaporation forms as a function of laser pulse energy.

**Figure 4:**
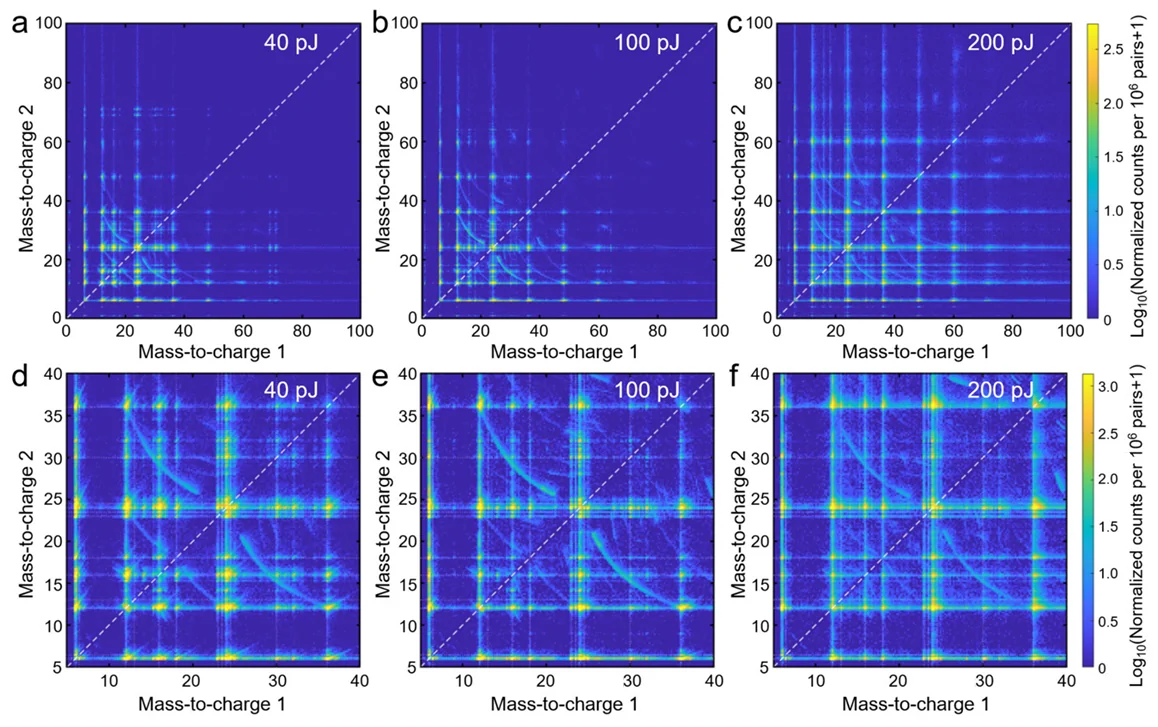
Normalized correlation histograms of TiC_x_ acquired at different laser energies. (**a**–**c**) Full mass-to-charge range for 40, 100 and 200 pJ. (**d**–**f**) Enlarged views showing the 0–40 Da regions.

**Figure 5:**
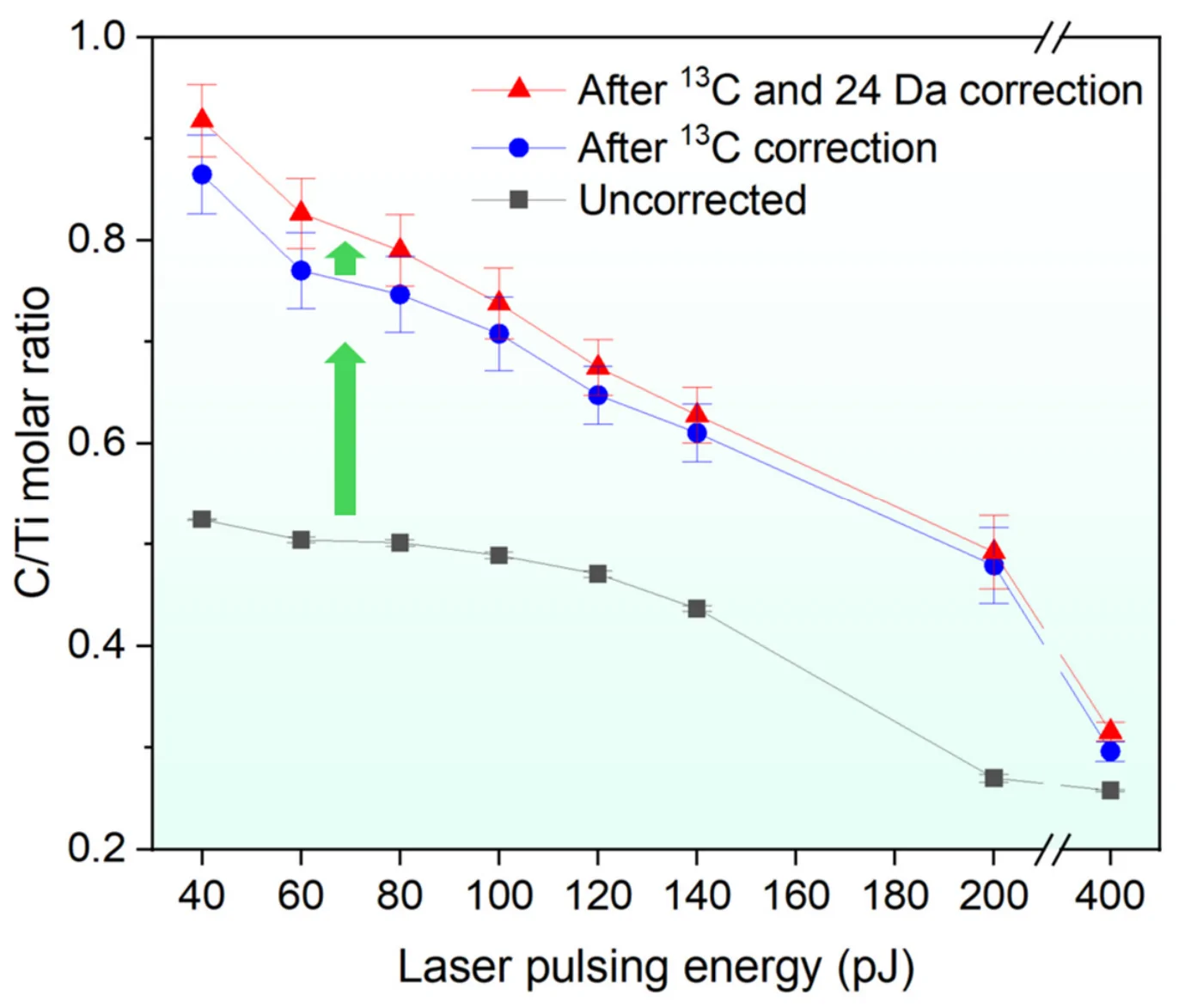
Uncorrected and corrected C/Ti ratios of TiC_x_ as a function of laser pulse energy. Error bars represent only the uncertainty propagated from the reported ^13^C natural abundance and do not include uncertainties associated with peak ranging, the 24 Da correction or tip-to-tip variability.

**Table 1:** Direct APT-measured compositions of TiC_x_ obtained at different laser pulse energies (at.%).

	Ti	C	O	N	Al
40 pJ	64.29	33.72	1.62	0.34	0.03
60 pJ	65.03	32.80	1.84	0.29	0.04
80 pJ	65.15	32.67	1.79	0.30	0.09
100 pJ	65.65	32.11	1.86	0.29	0.09
120 pJ	66.44	31.28	1.89	0.30	0.09
140 pJ	67.96	29.69	1.98	0.26	0.11
200 pJ	76.64	20.70	2.30	0.25	0.11
400 pJ	78.18	20.17	1.19	0.36	0.10

## Data Availability

The original contributions presented in this study are included in the article/Supplementary Material. Further inquiries can be directed to the corresponding author.

## Supplementary Materials

The following supporting information can be downloaded at https://www.mdpi.com/article/10.3390/ma19194055/s1. Table S1: APT dataset information acquired at different laser pulse energies; Figure S1: APT mass spectra of TiCx obtained at laser pulse energies of (**a**) 80 pJ, (**b**) 100 pJ, (**c**) 120 pJ, (**d**) 140 pJ and (**e**) 200 pJ; Note S1: Carbon loss correction procedure. References [28,40] are cited in the supplementary materials.
