# Supplementary material for: Reliable Atom Probe Quantification of Carbon in Non-Stoichiometric TiCx: Effects of Laser Energy and Correction of Carbon Loss
Source: Materials (Basel). 2026 Sep 23;19(19):4055. doi: 10.3390/ma19194055 (PMC13665968; doi:10.3390/ma19194055)
Supplement: Supplementary file 1 [file materials-19-04055-s001.zip › materials-4502552-supplementary.pdf]

Supplementary

Table S1. APT dataset information acquired at different laser pulse energies.

Laser energy (pJ)	40	60	80	100	120	140	200	400
Total ions (million)	5.0	5.0	5.0	5.0	5.0	5.0	6.0	6.0
Single (%)	59.8	65.0	67.8	71.3	74.0	76.1	83.6	91.4
Multiple (%)	40.0	34.7	32.0	28.4	25.7	23.6	16.0	8.2
Partial (%)	0.3	0.3	0.3	0.3	0.3	0.3	0.3	0.4
Volt./bowl corr. peak (Da)	24	24	24	24	24	24	24	24
(M/ΔM) for $^{48}Ti^{2+}$	207.3	215.8	376.5	613.0	700.4	711.7	733.2	590.1
Background (ppm/ns)	17.99	16.59	19.36	20.66	21.02	21.05	23.52	9.64

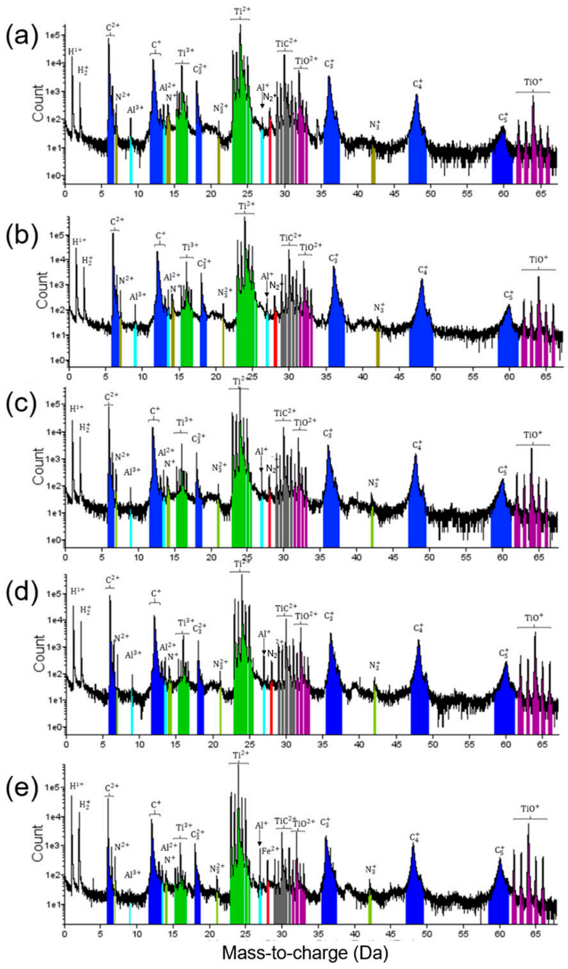

Figure S1. APT mass spectra of TiC_x obtained at laser pulse energies of (a) 80 pJ, (b) 100 pJ, (c) 120 pJ, (d) 140 pJ and (e) 200 pJ.

Note S1 Carbon loss correction procedure

The in-house program used background-corrected total, single-hit and multiple-hit mass spectra exported from IVAS 6.3. Ion counts were integrated over the mass-to-charge intervals defined in the same ranging file for all datasets. No additional peak-profile fitting was performed.

S1.1 ^{13}C isotope-abundance correction

The counts of $^{12}C^{2+}$ at 6 Da and $^{12}C^{+}$ at 12 Da were estimated from the corresponding $^{13}C^{2+}$ and $^{13}C^{+}$ peaks at 6.5 and 13 Da, respectively:

$$N(^{12}\mathrm{C}^{q+}) = N(^{13}\mathrm{C}^{q+}) \times (100 - f_{13})/f_{13}, \quad q = 1, 2 \qquad \text{(S1)}$$

Here, f_{13} = 1.07% is the natural abundance of ^{13}C[R1]. The central isotope-abundance correction factor was

$$R_0 = \frac{100 - f_{13}}{f_{13}} = \frac{100 - 1.07}{1.07} = 92.46 \qquad \text{(S2)}$$

The reported uncertainty in the ^{13}C abundance, Δf_{13} = 0.08%[R1], was propagated using lower and upper correction factors:

$$R_{low} = \frac{100 - f_{13} - \Delta f_{13}}{f_{13} + \Delta f_{13}} = 85.96 \qquad \text{(S3)}$$

$$R_{up} = \frac{100 - f_{13} + \Delta f_{13}}{f_{13} - \Delta f_{13}} = 100.01 \qquad \text{(S4)}$$

The lower and upper corrected ^{12}C counts were calculated as

$$N_{low}(^{12}\mathrm{C}^{q+}) = R_{low} \times N(^{13}\mathrm{C}^{q+}) \qquad \text{(S5)}$$

$$N_{up}(^{12}\mathrm{C}^{q+}) = R_{up} \times N(^{13}\mathrm{C}^{q+}) \qquad \text{(S6)}$$

S1.2 Correction of the 24 Da peak

The 24 Da correction followed the method proposed by Angseryd et al. in [R2]. The peaks at 23 and 23.5 Da were assigned to the non-overlapped $^{46}Ti^{2+}$ and $^{47}Ti^{2+}$

isotopes, respectively. Assuming that the relative Ti-isotope distribution should be the same in the single-hit and multiple-hit spectra in the absence of detector loss, the expected multiple-hit count at 24 Da was calculated as

$$N_{\text{expected}}(24, m) = N(24, s) \times [N(23, m) + N(23.5, m)]/[N(23, s) + N(23.5, s)]$$

(S7)

Here, s and m denote the single-hit and multiple-hit spectra, respectively. The number of incompletely registered ions at 24 Da was estimated as

$$\Delta N_{24} = N_{\text{expected}}(24, m) - N(24, \mathrm{m}) \qquad \text{(S8)}$$

The recovered signal was assigned to C_2^+. Therefore, two carbon atoms were added for each recovered ion:

$$\Delta N_C = 2\Delta N_{24} \qquad \text{(S9)}$$

S1.3 Corrected composition

For each element i, the corrected atom count was calculated as

$$A_i = \sum_r v_{i,r} \times N_r^{corr} \qquad \text{(S10)}$$

where N_r^{corr} is the corrected count of ion species r and $v_{i,r}$ is the number of atoms of element i contained in that ion species. The corrected atomic concentration was calculated as

$$c_i = A_i / \sum_j A_j \times 100\% \qquad \text{(S11)}$$

The corrected C/Ti ratio was calculated directly from the corrected carbon and titanium atom counts:

$$\mathrm{C/Ti}_{corr} = A_{\mathrm{C}}/A_{\mathrm{Ti}} \qquad \text{(S12)}$$

The central isotope-corrected composition was calculated using R_0, while R_{low}

and R_{up} were used to determine the uncertainty bounds.

[R1] J.K. Böhlke, J.R. de Laeter, P. De Bièvre, H. Hidaka, H.S. Peiser, K.J.R. Rosman, P.D.P. Taylor, Isotopic compositions of the elements, 2001, *Journal of Physical and Chemical Reference Data* 34 (2005) 57–67.

[R2] J. Angseryd, F. Liu, H.O. Andren, S.S. Gerstl, M. Thuvander, Quantitative APT analysis of Ti(C,N), Ultramicroscopy 111(6) (2011) 609-614.
